# Yield performance, mineral profile, and nitrate content in a selection of seventeen microgreen species

**DOI:** 10.3389/fpls.2023.1220691

**Published:** 2023-07-20

**Authors:** Francesco Di Gioia, Jason C. Hong, Cristina Pisani, Spyridon A. Petropoulos, Jihne Bai, Erin N. Rosskopf

**Affiliations:** ^1^ Department of Plant Science, The Pennsylvania State University, University Park, PA, United States; ^2^ U.S. Department of Agriculture (USDA), Agricultural Research Service (ARS), U.S. Horticultural Research Laboratory, Fort Pierce, FL, United States; ^3^ U.S. Department of Agriculture (USDA), Agricultural Research Service (ARS), Southeastern Fruit and Tree Nut Research Station, Byron, GA, United States; ^4^ Department of Agriculture Crop Production and Rural Environment, University of Thessaly, Volos, Greece

**Keywords:** Amaranthaceae, Amaryllidaceae, Asteraceae, Boraginaceae, Brassicaceae, Chenopodiaceae, ionome, Lamiaceae

## Abstract

**Introduction:**

Originally regarded as garnish greens, microgreens are increasingly valued for their nutritional profile, including their mineral content.

**Methods:**

A study was conducted under controlled environmental conditions utilizing a selection of seventeen microgreen species belonging to seven different botanical families to investigate the genetic variation of macro- and micro-minerals and nitrate (NO_3_^-^) content. Plants were grown in a soilless system using a natural fiber mat as the substrate. After germination, microgreens were fertigated with a modified half-strength Hoagland solution prepared using deionized water and without adding microelements. At harvest (10 to 19 days after sowing, based on the species), yield components were measured and dry tissue samples were analyzed for the concentration of total nitrogen (N), NO_3_^-^, P, K, Ca, Mg, S, Na, Fe, Zn, Mn, Cu, and B.

**Results and discussion:**

Genotypic variations were observed for all of the examined parameters. Nitrogen and K were the principal macronutrients accounting for 38.4% and 33.8% of the total macro-minerals concentration, respectively, followed in order by Ca, P, S, and Mg. Except for sunflower (*Helianthus annuus* L.), all the tested species accumulated high (1,000-2,500 mg kg^-1^ FW) or very high (>2,500 mg kg^-1^ FW) NO_3_^-^ levels. Eight of the studied species had a K concentration above 300 mg 100 g^-1^ FW and could be considered as a good dietary source of K. On the other hand, scallion (*Allium fistulosum* L.), red cabbage (*Brassica oleracea* L. var. *capitata*), amaranth (*Amaranthus tricolor* L.), and Genovese basil (*Ocinum basilicum* L.) microgreens were a good source of Ca. Among micro-minerals, the most abundant was Fe followed by Zn, Mn, B, and Cu. Sunflower, scallion, and shiso (*Perilla frutescens* (L.) Britton) were a good source of Cu. Moreover, sunflower was a good source of Zn, whereas none of the other species examined could be considered a good source of Fe and Zn, suggesting that supplementary fertilization may be required to biofortify microgreens with essential microminerals. In conclusion, the tested microgreens can be a good source of minerals showing a high potential to address different dietary needs; however, their yield potential and mineral profile are largely determined by the genotype.

## 1 Introduction

Microgreens are increasingly recognized as an emerging category of horticultural products with distinctive characteristics compared to sprouts, baby leaf, and standard vegetable products (Di Gioia et al., 2017c). Initially regarded as garnishes, today microgreens continue to attract consumers primarily for their interesting nutritional profiles (Kyriacou et al., 2016). A growing body of literature is highlighting the potential of microgreens as a source of essential minerals, vitamins, and other bioactive compounds considered beneficial for human health (Kyriacou et al., 2019; Ghoora et al., 2020; Kyriacou et al., 2021a; Teng et al., 2023).

Given their rich nutritional profile, microgreens have gained the appellative of superfood, and have been proposed as a potential nutrition security resource (Di Gioia et al., 2021). As nutrient-dense young seedlings, produced in a relatively short time, in limited space, and using minimum inputs, microgreens could be used easily to diversify diets and address malnutrition issues affecting large sections of the world population, especially in areas affected by shortages of fresh vegetables due to climate change, emergencies, and/or human conflicts. They can also be considered an ideal target crop for agronomic biofortification to produce functional greens fortified with essential micronutrients (Di Gioia et al., 2019a; Germ et al., 2019; Puccinelli et al., 2021; Kathi et al., 2023).

Another aspect that makes microgreens particularly interesting, both from a commercial and a nutritional perspective, is the variety of species that can be used for their production. Except for species that are toxic at the seedling stage, as is the case for *Solanaceae* crops, a wide range of vegetables, cereals, pseudocereals, legumes, herbs, and wild edible species may be used to produce microgreens (Kyriacou et al., 2016; Di Gioia et al., 2017c; Benincasa et al., 2019). The opportunity to use a large variety of species is interesting due to the diversity of microgreen colors, flavors, textures, and aesthetic quality traits that may be obtained, as well as for the diverse nutritional profile associated with genetic variability. Using a wide range of species and agrobiodiversity resources, microgreens can in fact contribute to diet diversification and address the needs of consumers that have dietary restrictions and defined mineral needs.

Despite the availability in seed catalogues of a wide array of species suitable for microgreen production, most of the literature currently available on the mineral profile of microgreens is focused primarily on taxa belonging to the *Brassicaceae* family (Kyriacou et al., 2016; Xiao et al., 2016; Kyriacou et al., 2019; Kyriacou et al., 2021a). Other taxa commonly grown as microgreens include *Amaranthaceae*, *Amaryllidaceae*, *Apiaceae*, *Asteraceae*, *Chenopodiaceae*, *Fabaceae*, *Lamiaceae*, *Poaceae*, and *Polygonaceae*. Although some literature is available on the mineral profile of microgreens belonging to specific botanical families and taxa (Corrado et al., 2022; Giordano et al., 2022), a limited number of studies have investigated the genetic variability between taxa belonging to different botanical families.

Investigating the genetic variation of the microgreens ionome within and between different botanical families under controlled conditions may allow classification of microgreen species for their capacity to accumulate specific macro- or micro-minerals and to identify species or taxa with a mineral profile of particular interest for consumers that have specific dietary needs or are particularly suitable to address mineral malnutrition issues such as Ca, Fe, or Zn deficiency. This information may also be useful in identifying microgreen species suitable as specific mineral biofortification targets (Di Gioia et al., 2019a; Poudel et al., 2023a).

In addition to the content of essential minerals, microgreens, like other leafy greens, can accumulate relatively high levels of nitrates (NO_3_). From one perspective, NO_3_^-^ are considered antinutrients, potentially harmful for consumers, and are therefore subject to regulations and commercial agreements that set limits in their content (Di Gioia et al., 2013; Kyriacou et al., 2021b); on the other hand, NO_3_^-^ are considered potentially beneficial for other consumers such as athletes who may improve their performance through NO_3_^-^ supplementation (Hord et al., 2009; Hoon et al., 2013). Despite their potential effects on human health, limited information is available on the genetic variation of NO_3_^-^ content in microgreens. The potential mineral profile and NO_3_^-^ content of microgreens, while determined genetically, is largely affected by the quantity of nutrients available through the growing media or in the nutrient solution used and is highly influenced by other environmental factors such as temperature, light intensity and quality (Samuoliene et al., 2013; Di Gioia et al., 2017a; Kyriacou et al., 2019; Bulgari et al., 2021; Kyriacou et al., 2021b; Poudel et al., 2023b). Therefore, comparing different species under the same agronomic and environmental conditions is critical to identify possible genetic variations. Many commercial microgreen growers and consumers producing microgreens for self-consumption claim that microgreens do not require any fertilization, especially for microminerals, because most of the micro-nutrients needed for adequate seedling growth are already available in the seeds. From this perspective, it is interesting to investigate the micromineral content of microgreens without the application of micromineral fertilization. Under such controlled conditions, the micronutrient mineral profile may be defined and potentially limited by the quantity of nutrients available in the seeds of each species. For this purpose, a study was conducted under controlled conditions to investigate the yield performance, ionome, and NO_3_^-^ content variation within a selection of seventeen microgreen species belonging to seven different botanical families. We hypothesize that, under controlled environmental conditions, yield performance, mineral content, and NO_3_^-^ accumulation are largely determined by the microgreen’s genotype and species belonging to different botanical families will perform differently. To test this hypothesis, plants of different species were grown at the same time, under controlled environmental conditions, in a soilless system, using natural fiber mats as the substrate, and a modified half-strength Hoagland nutrient solution prepared using deionized water and without adding any microminerals. The studied species were selected as they are among the most popular species grown as microgreens in the US and our aim was to identify those presenting higher nutritional value in terms of mineral content. The results of the present study will advance our understanding of the genetic variation of the mineral profile of microgreens in association with their yield performance and will contribute to define the role microgreens may play as a source of macro- and micro-minerals and NO_3_^-^ in our daily diet, without the implementation of biofortification strategies.

## 2 Materials and methods

### 2.1 Experimental site, plant material, and growing conditions

The following study was conducted at the greenhouse facility of the United States Horticultural Research Laboratory of the US Department of Agriculture, Agricultural Research Service located in Fort Pierce, FL, USA (27°25′38′′N, 80°24′35′′W; 5 m a.s.l.), in a 149 m^2^ polycarbonate-covered greenhouse with cooling system and forced air ventilation. A selection of seventeen microgreen species belonging to the families of *Amaranthaceae* [amaranth (*Amaranthus tricolor* L.)], *Amaryllidaceae* [scallion (*Allium fistulosum* L.)], *Asteraceae* [sunflower (*Helianthus annuus* L.)], *Boraginaceae* [borage (*Borago officinalis* L.)], *Brassicaceae* [arugula (*Eruca sativa* (Mill.) Thell.), broccoli (*Brassica oleracea* L. var. *italica* Plenck), red cabbage (*Brassica oleracea* L. var. *capitata*), cress (*Lepidium sativum* L.), kale (*Brassica napus* L. var. *pabularia* (DC.)), mizuna (*Brassica rapa* L. var. *japonica*), mustard (*Brassia juncea* (L.) Czern.), radish (*Raphanus sativus* L.)], *Chenopodiaceae* [beet (*Beta vulgaris* L.)], and *Lamiaceae* [red basil (*Ocinum basilicum* L.), green basil (*Ocinum basilicum* L.), shiso (*Perilla frutescens* (L.) Britton), and lemon balm (*Melissa officinalis* L.)] were evaluated. Seeds of all the species were purchased from Johnny’s Selected Seeds (Fairfield, Maine, USA). Commercial and scientific name, 1000-seed weight, percentage of seed germination, seeding density, and days from seeding to harvest of each species are presented in **Table 1**. All microgreen species were seeded on May 11, 2018, and were grown under natural light in a soilless system constructed with growing channels (Cropking Inc., Lodi, OH, USA) 0.25 m wide and 1.4 m long placed on growing benches with a slope of approximately 7%. Each channel constituted an experimental unit hosting a single species grown at the defined density on three BioStrate-Felt (Cropking Inc., Lodi, OH, USA) growing mats (25 × 25 cm = 625 cm^2^). Three growing channels were seeded for each species. Species were distributed according to a completely randomized block design with three replications.

**Table 1:** Botanical family, scientific and common name, seed quality, seed density, and growth cycle of the selected seventeen microgreen species.

Botanical family	Scientific name	Common name	1000-seed weight (g)	Germination percentage (%)	Seed density (seed cm^-2^)	Days from seeding to harvest
*Amaranthaceae*	*Amaranthus tricolor* L.	‘Garnet red’ amaranth	0.779	98	7.5	18
*Amaryllidaceae*	*Allium fistulosum* L.	Scallion	2.060	95	3.0	14
*Asteraceae*	*Helianthus annuus* L.	‘Black oil’ sunflower	58.295	90	1.0	10
*Boraginaceae*	*Borago officinalis* L.	Borage	19.039	99	1.0	12
*Brassicaceae*	*Eruca sativa* (Mill.) Thell.	Arugula	1.505	93	4.0	14
	*Brassica oleracea* L. var. *italica* Plenck	Broccoli	3.732	97	2.7	11
	*Brassica oleracea* L. var. *capitata*	Red cabbage	3.454	93	2.3	13
	*Lepidium sativum* L.	Cressida Cress	2.570	92	3.0	11
	*Brassica napus* L. var. *pabularia* (DC.)	‘Red Russian’ kale	3.830	98	3.0	11
	*Brassica rapa* L. var. *japonica*	Mizuna ‘America’	2.025	95	3.0	14
	*Brassia juncea* (L.) Czern.	‘Garnet giant’ mustard	1.994	97	3.0	12
	*Raphanus sativus* L.	‘Red arrow’ radish	7.748	98	2.0	10
*Chenopodiaceae*	*Beta vulgari*s L.	‘Bull’s blood’ beet	17.658	93	1.0	18
*Lamiaceae*	*Ocinum basilicum* L.	‘Dark opal’ basil (Red)	1.574	79	2.0	18
	*Ocinum basilicum* L.	‘Genovese’ basil (Green)	1.623	91	2.0	18
	*Perilla frutescens* (L.) Britton	Britton shiso	3.106	93	2.0	18
	*Melissa officinalis* L.	Lemon balm	0.669	86	3.0	19

After seeding, all the species were irrigated manually with deionized water by means of a nursery water nozzle and were covered with a white-on-black polyethylene film until complete germination. The film was removed upon complete seed germination of each species after daily inspection, and seedlings were then grown under sunlight and fertigated by subirrigation with a film of nutrient solution running through each channel and delivered on the upper end of the channel through an orchard tube line with three pressure-compensated drippers (each with a delivery rate of 4.0 L h^-1^) per channel. The nutrient solution was prepared with deionized water and contained (mM): 7.5 N (6.97 NO_3_-N and 0.53 NH_4_-N), 0.5 P, 3.0 K, 2.5 Ca, 1.0 Mg, and 1.8 S, resulting in an EC of 1.3 dSm^-1^ and pH 6.2. No micronutrients were added to the nutrient solution to evaluate the quantity of micronutrients provided from the seeds of each species and test whether the seeds can provide enough micronutrients, considering the short growing period of microgreens. With an open cycle management system, fertigation events were scheduled daily through a timer with multiple events of 1 minute to assure a minimum drainage fraction of 20% for all the species. During the experiment, minimum and maximum air temperatures of the greenhouse were set at 20 and 28 °C, respectively, resulting in an average air temperature of 25.4 °C. The relative humidity averaged 76% and ranged between a minimum of 43% and a maximum of 97%. Daily solar radiation was on average 262 W/m^2^ and ranged between a minimum average of 134 W/m^2^ and a maximum average of 324 W/m^2^.

### 2.2 Microgreens harvest, yield assessment, sample preparation, and mineral analyses

Microgreens of each species were harvested upon achievement of the commercial harvesting stage which corresponded to the presence of fully expanded, colored, and turgid cotyledons and the initial growth of true leaves.

All microgreens were harvested by cutting the seedlings just above the surface of the growing mat with clean cutter blades. After cutting, microgreens were weighed to determine the fresh yield (g m^-2^) and a pre-marked 10 × 10 cm area at the center of each growing mat was used to count the number of shoots and measure the mean shoot fresh weight (mg shoot^-1^). Dry matter content (g kg^-1^ FW) was determined in samples of approximately 150 g dried until constant weight at 65°C in a forced-draught oven. Dried plant tissue samples were ground through a mill to pass through a 1.0 mm sieve and were used to determine the concentration of total N, NO_3_-N, P, K, Ca, Mg, S, Na, Fe, Zn, Cu, Mn, and B.

Total N concentration was determined by combustion according to the Dumas method using an auto‐analyzer (NC Soil Flash EA1112, CE Elantech Inc., Lakewod, NJ, USA) as described by Vecchia et al. (2020). The concentration of NO_3_-N was determined by ion chromatography (model QIC; Dionex Corp. Sunnyvale, CA, USA) after extraction from dry samples of 0.5 g with 20 mL of sodium carbonate (3.5 mmol L^−1^) and sodium bicarbonate (1.0 mmol L^−1^) solution following the procedure described by Di Gioia et al. (2017b). The concentration of P, K, Ca, Mg, S, Na, Fe, Zn, Cu, Mn, and B was determined by inductively coupled plasma atomic emission spectrometry (ICP‐AES; iCAP 6500, Thermo Scientific, Waltham, MA, USA) after microwave‐assisted digestion (MARS Express, CEM Corp., Matthews, NC, USA) according to U.S. EPA method 3052 (USEPA, 1997). For all the plant tissue analyses, quality control standards and distilled‐deionized water blanks were used to ensure that the ion chromatography and the ICP‐AES system were operating properly.

### 2.3 Statistical analysis

The effect of the genotype on all of the measured parameters was evaluated by performing the analysis of variance (ANOVA) with the GLM procedure of SAS Version 9.4 software (SAS Institute, Cary, NC, USA). When significant differences were observed, means were compared *via* Duncan’s multiple range test at *P*=0.05. Pearson correlation coefficients between average seed weight and mean shoot fresh weight and between total N and NO_3_-N concentration of all of the species examined were calculated using the CORR procedure of SAS. Linear regression analysis was performed using the SAS REG procedure to estimate the relationship between average seed weight and mean shoot fresh weight and between total N and NO_3_-N concentration across all of the species tested. Before conducting the principal component analysis (PCA), Kaiser-Meyer-Olkin (KMO) and Bartlett’s sphericity test were performed using the FACTOR procedure of SAS to measure the sampling adequacy. The KMO value was 0.65, and the Bartlett’s sphericity test was significant (*P* < 0.0001; χ^2 =^ 896 df =120) suggesting that the dataset met the criteria for factor analysis and for using PCA as a data reduction technique. The PCA was conducted using the PRINCOMP procedure of SAS. The PCA bidimensional plots were visualized using the software PAST4.04. Before carrying out PCA, means were standardized [(x-mean)/standard deviation] as described by Petropoulos et al. (2019).

## 3 Results and discussion

### 3.1 Microgreens harvesting time, yield, and quality

Substantial differences were observed among the tested microgreen species in terms of harvesting time, yield, and quality. Harvesting time measured in days after sowing (DAS) varied between 10 and 19 days. As reported in **Table 1**, sunflower and radish were harvested at 10 DAS; broccoli, cress, and kale were harvested at 11 DAS; borage and mustard at 12 DAS; red cabbage at 13 DAS; scallion, arugula and mizuna at 14 DAS; amaranth, beet, shiso, ‘Dark opal’ and ‘Genovese’ basil at 18 DAS; and lemon balm at 19 DAS.

**Figure 1:**
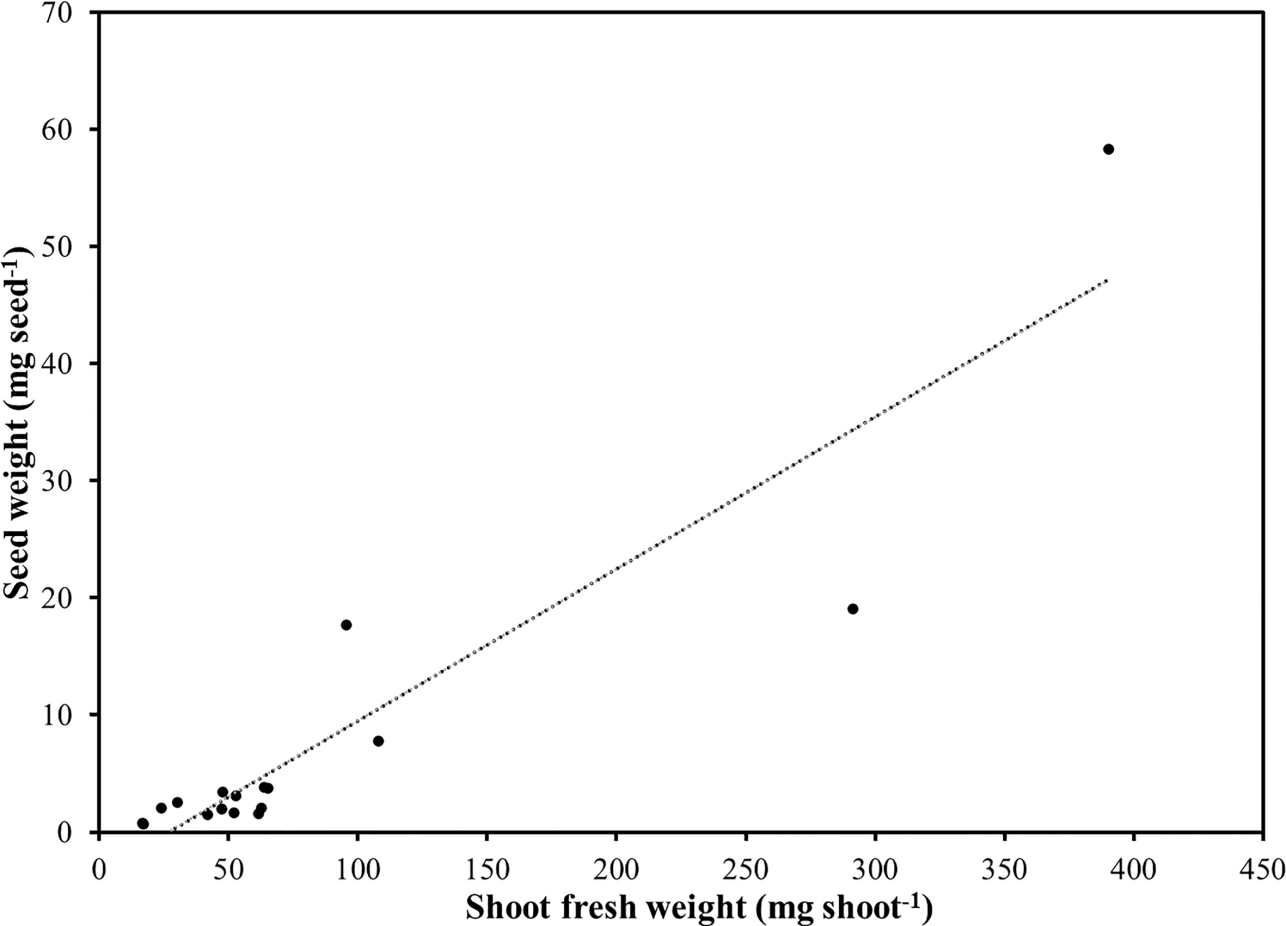
Relationship between average seed weight and shoot fresh weight in seventeen selected species of microgreens.

Fresh yield was, on average, 1,175.7 g m^-2^ and ranged from 409.3 g m^-2^ for lemon balm up to 2,258.8 g m^-2^ in the case of radish (**Table 2**). Such yield variability could be explained not only by the different seeding density and harvesting time but also by differences in seedling morphology at harvest among the species (Di Gioia and Santamaria, 2015). Following radish, borage and sunflower shoots provided the highest fresh yield per unit area. Excluding radish, *Brassicaceae* had intermediate fresh yield values ranging from 995 g m^-2^ for cress to 1,461 g m^-2^ for broccoli. Intermediate fresh yield values were also observed in the case of red basil, green basil, and shiso; whereas relatively low fresh yield values were obtained in the case of amaranth, beet, scallion, and lemon balm which were characterized by smaller shoots and/or relatively low germination rates. Shoot fresh weight was also highly variable and ranged from a minimum of 16.7 and 17.1 mg, in the case of amaranth and lemon balm, up to a maximum of 390.4 mg per shoot in the case of sunflower. Following sunflower, borage, radish, and beet were characterized as having higher single-shoot fresh weight as compared to all the of other greens. *Lamiaceae* and the remaining *Brassicaceae* species were characterized by intermediate single-shoot FW. A positive correlation (r=0.92; *P* < 0.0001) was observed between seed weight and shoot mean fresh weight for all the examined species, suggesting that, although influenced by seeding density and harvesting time, the shoot FW is largely determined by the genotype and by the initial size of seeds. The relationship between seed weight and shoot FW was linear with an adjusted R^2^ of 0.84 (**Figure 1**). Estimated slope was 0.1296 and was significant (*P* < 0.0001), while the intercept was not significantly different from zero.

**Table 2:** Fresh yield, single shoot fresh weight, and dry matter content of seventeen microgreen species.^1^

Genotype	Fresh yield	Shoot FW	Dry matter
	g m^-2^	mg shoot^-1^	g kg^-1^ FW
‘Garnet red’ amaranth	897.2 ± 15.0 h	16.69 ± 1.6 h	64.05 ± 1.1 c
Scallion	571.5 ± 21.0 il	23.86 ± 0.4 gh	89.06 ± 1.7 a
‘Black oil’ sunflower	1656.6 ± 42.5 bc	390.37 ± 16.0 a	94.34 ± 2.5 a
Borage	1766.8 ± 61.8 b	291.41 ± 16.2 b	44.07 ± 0.7 efgh
Arugula	1318.7 ± 53.7 de	41.9 ± 0.5 efg	46.19 ± 0.3 defg
Broccoli	1461.1 ± 59.8 cd	65.29 ± 5.9 d	38.55 ± 2.7 h
Red cabbage	1251.0 ± 44.4 def	61.5 ± 1.6 de	50.07 ± 0.9 de
Cressida Cress	994.7 ± 30.4 gh	30.22 ± 0.7 fgh	40.64 ± 1.2 gh
‘Red Russian’ kale	1444.1 ± 30.5 d	62.62 ± 3.2 de	44.04 ± 2.1 efgh
Mizuna ‘America’	1211.7 ± 19.4 efg	47.59 ± 0.4 def	52.82 ± 0.4 d
‘Garnet giant’ mustard	1081.5 ± 55.7 gh	47.28 ± 3.3 def	48.41 ± 0.9 def
‘Red arrow’ radish	2258.8 ± 40.9 a	107.86 ± 10.2 c	41.32 ± 1.7 fgh
‘Bull’s blood’ beet	698.3 ± 24.8 i	95.46 ± 4.7 c	61.71 ± 1.4 c
‘Dark opal’ basil	1024.3 ± 158.0 gh	63.81 ± 6.1 de	43.26 ± 2.6 efgh
‘Genovese’ basil	931.0 ± 172.7 h	52.24 ± 11.5 def	65.03 ± 6.8 c
Britton Shiso	1009.9 ± 18.1 gh	52.94 ± 1.9 de	68.55 ± 1.0 c
Lemon Balm	409.3 ± 30.4 l	17.12 ± 0.2 h	75.34 ± 0.9 b
*P-value*	*0.0001*	*0.0001*	*0.0001*

^1^ Data reported are means ± standard error of three replications. Means followed by different letters within each column are significantly different (P < 0.05) by Duncan’s multiple range test. Microgreens were grown under controlled environmental conditions in a polycarbonate greenhouse located in Fort Pierce, FL, USA. Each species was grown in separate growing channels using BioStrate-Felt growing mats and were fertigated with a standard nutrient solution containing only macronutrients.

Observed fresh yield values were similar to those reported in other studies where microgreens were grown with similar seed densities and harvested at the fully expanded cotyledon growth stage (Lee et al., 2004; Murphy and Pill, 2010; Bulgari et al., 2017; Di Gioia et al., 2017a). However, the recorded fresh yield values were lower when compared to values observed in studies conducted using higher seed densities and/or in which greens were harvested at a more advanced growth stage (Murphy et al., 2010; Kyriacou et al., 2019). Broccoli fresh yield was similar to that of *Brassica rapa* L. grown in recycled textile fiber or jute-kenaf fiber mat (Di Gioia et al., 2017a). Arugula fresh yield was similar to that observed for the same species grown in peat at lower seed density but harvested at similar stage (Murphy and Pill, 2010), and slightly lower than that reported for arugula microgreens grown in a floating mat system (Bulgari et al., 2017). Beet fresh yield observed in this study was within the range reported for red beet and Swiss chard by Lee et al. (2004), but was lower when compared to values reported by Murphy et al. (2010) for pre-germinated beet seeds grown either in a peat-lite mix or in a hydroponic system. In the case of basil, fresh yield values were comparable to those reported by Bulgari et al. (2017) for basil microgreens grown in a floating or deep-water culture system. To the best of our knowledge, limited or no yield data information is available in the literature for the other species of microgreens examined in this study.

Dry matter content ranged between 38.55 g kg^-1^ FW in broccoli and 94.34 g kg^-1^ FW in the case of sunflower and was on average 56.90 g kg^-1^ FW. Similar dry matter variation ranges were observed by Kyriacou et al. (2019) and Xiao et al. (2012) who examined thirteen and twenty-five species of microgreens, respectively. All of the tested *Brassicaceae* species contained relatively low levels of dry matter as compared to the other species, with the exception of borage and red basil. Observed dry matter values of the *Brassicaceae* species were lower when compared to values observed by Xiao et al. (2016) from a selection of thirty *Brassicaceae* genotypes grown in a peat moss medium. Considering the contradictory results reported in the literature, it could be suggested that irrigation practices may greatly influence the water content of microgreens and the reduced levels of dry matter observed in this study could be explained by the supply of nutrient solution in excess to guarantee a minimum drainage fraction of 20%.

### 3.2 Variation in macrominerals, nitrate, and sodium concentration

**Figure 2:**
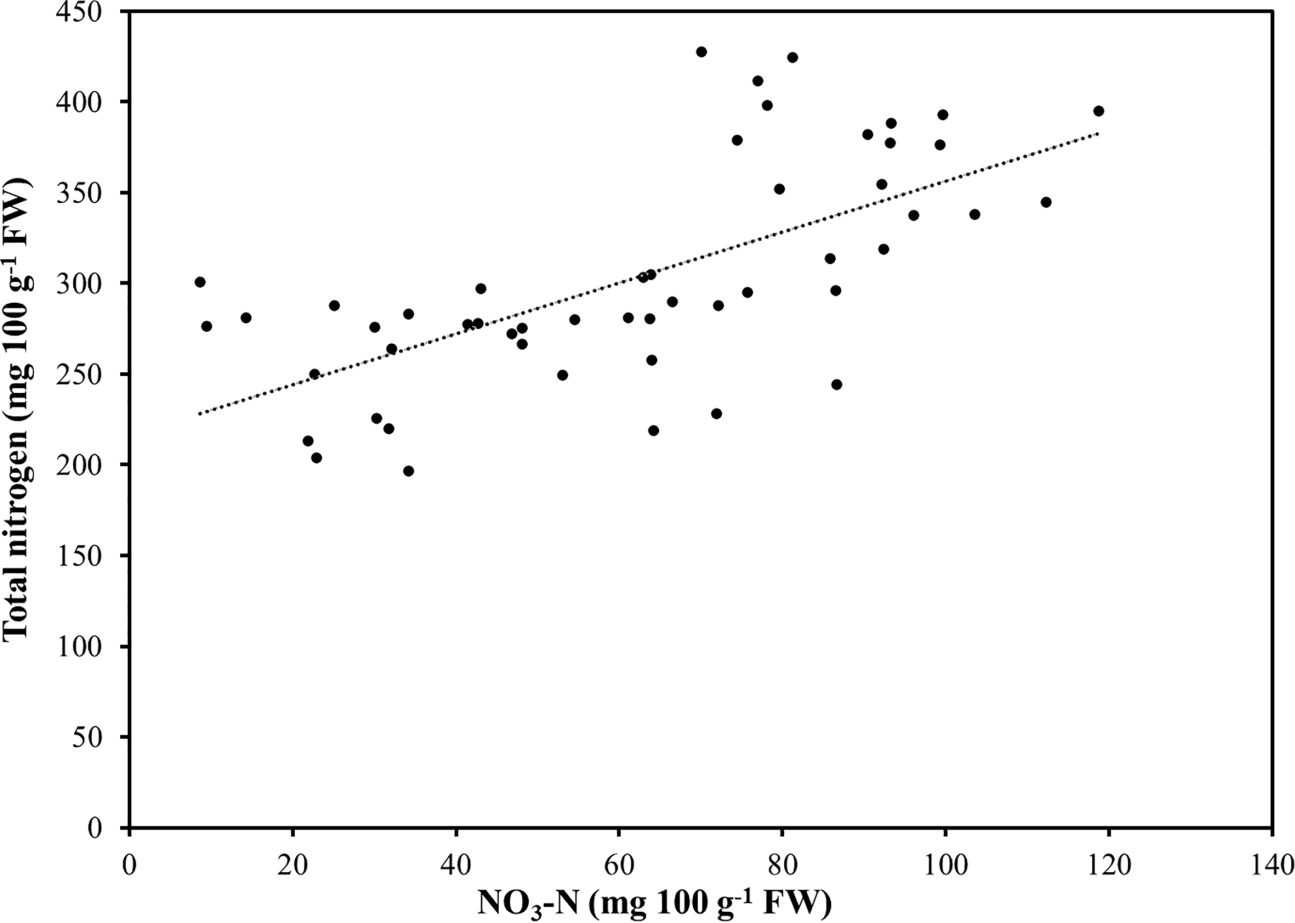
Relationship between total nitrogen and nitrate-N (NO_3_-N) in seventeen selected species of microgreens.

**Figure 3:**
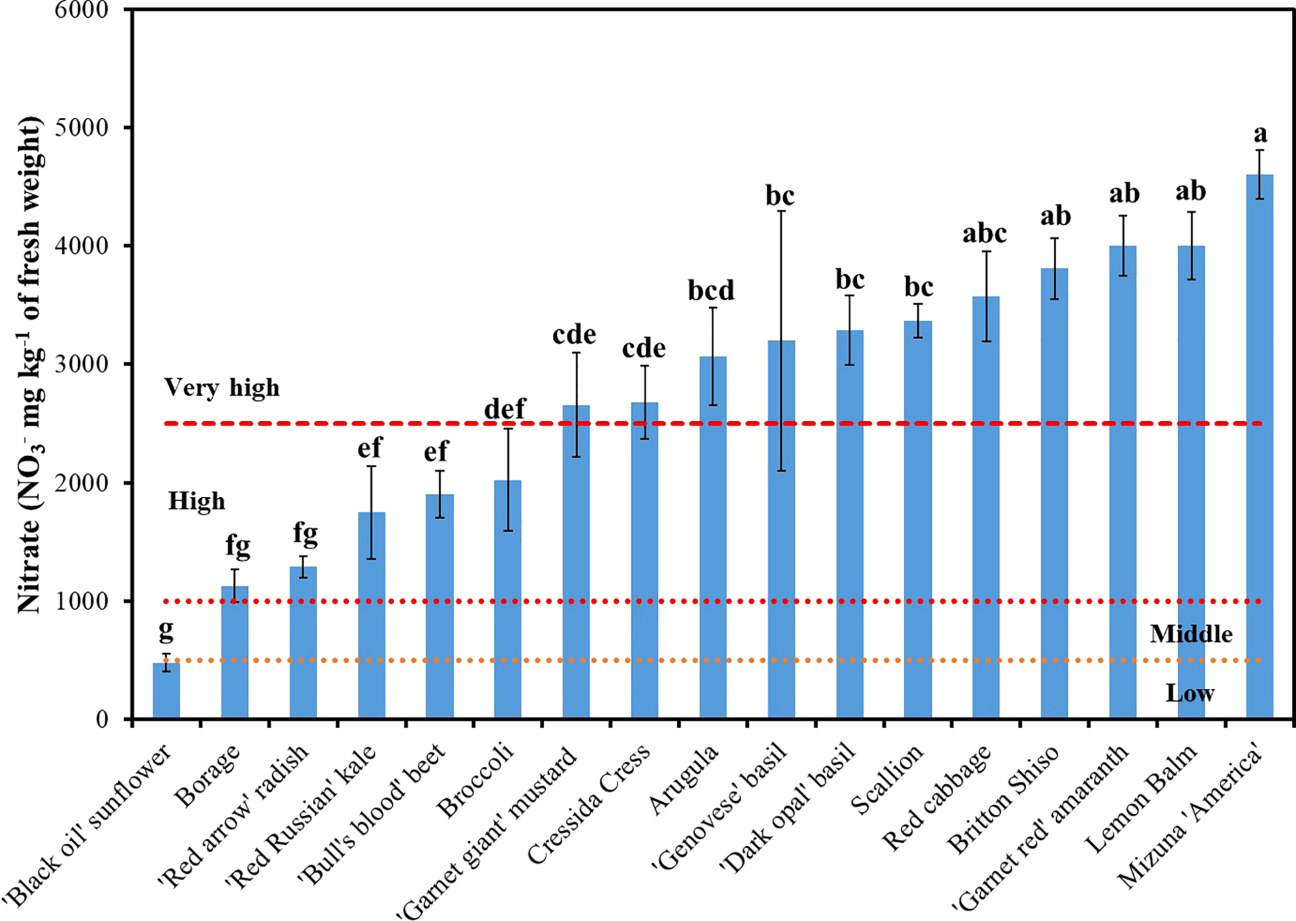
Variation of nitrate (NO_3_^-^) concentration among seventeen species of microgreens ranked within low (<500 mg kg^-1^), middle (500-1,000 mg kg^-1^), high (1,000-2,500 mg kg^-1^), and very high (>2,500 mg kg^-1^) NO_3_^-^ content on a fresh weight basis according to Di Gioia et al. (2013). Data reported are means of three replications. Vertical bars represent the mean ± standard error; means followed by different letters are significantly different (*P <*0.05) by Duncan’s multiple range test.

Substantial variations in macroelement concentrations were observed among the seventeen genotypes analyzed (**Table 3**). Nitrogen and K were the principal macronutrients accounting on average for 38.4% and 33.8% of the total macroelement concentration, respectively, followed in order by Ca, P, S, Mg, and Na, which accounted on average for 10.5%, 6.4%, 6.3%, 3.6% and 0.9% of the total macroelement concentration, respectively. The relative proportions of macroelements observed are consistent with the findings of previous studies which investigated the mineral composition of microgreens (Pinto et al., 2015; Xiao et al., 2016; Di Gioia et al., 2017a; Di Gioia et al., 2017c; Kyriacou et al., 2019). Reegarding the particular species, total N ranged between 212.3 and 421.3 mg 100 g^-1^ of FW in borage and scallion, respectively. Nitrate-N constituted, on average, 20.6% of the total N, ranging from a minimum of 3.8% in sunflower to a maximum of 45.1% of total N in red basil. Across all of the species examined, a positive correlation (r=0.66; *P* < 0.0001) was observed between total N and NO_3_-N suggesting that the concentration of total N is positively influenced by the NO_3_^-^ accumulation capacity of the genotypes examined. There was also a positive linear relationship between total N and NO_3_-N with an Adjusted R^2^ of 0.43 (**Figure 2**). Estimated slope and intercept values were 1.4 and 216.2, respectively, being significant at *P* < 0.0001. Expressed as NO_3_^-^ in mg kg^-1^ FW, the concentration of NO_3_^-^ was on average 2,753 mg kg^-1^ of FW and ranged from a minimum of 458.6 mg kg^-1^ of FW in sunflower up to 4,602 mg kg^-1^ of FW in mizuna (**Figure 3**). Based on the classification proposed by Di Gioia et al. (2013), eleven of the seventeen genotypes examined had a very high (>2,500 mg kg^-1^ FW) NO_3_^-^ concentration; whereas borage, radish, ‘Red Russian’ kale, beet, and broccoli had high (1,000-2,500 mg kg^-1^ FW) NO_3_^-^ concentration; and ‘Black oil’ sunflower had a low (<500 mg kg^-1^ FW) NO_3_^-^ concentration. These results are consistent with the findings of Di Gioia and Santamaria (2015) and Di Gioia et al. (2017c) who observed very high NO_3_^-^ accumulation levels, especially in *Brassicaceae* and basil supplied with N in the nutrient solution at the same level used in this study. Besides red and green basil, in the present study, lemon balm and Britton shiso belonging to the same botanical family, also accumulated very high levels of NO_3_^-^. A very high concentration of NO_3_^-^ was observed also in ‘Red garnet’ amaranth and scallion. Overall, the results of the present study are consistent with previous studies reporting that leafy vegetables belonging to the *Brassicaceae*, *Lamiaceae*, *Chenopodiaceae*, and *Amaranthaceae* families tend to accumulate high levels of NO_3_^-^ (Di Gioia et al., 2013), and also revealed that scallion, belonging to the *Amaryllidaceae* family, can accumulate high levels of NO_3_^-^ when harvested as a microgreen, unlike its mature counterpart (Chang et al., 2013). Similar variation in NO_3_^-^ concentrations was observed by Kyriacou et al. (2019) in a selection of thirteen genotypes belonging to *Apiaceae*, *Brassicaceae*, *Chenopodiaceae*, *Lamiaceae*, and *Malvaceae* harvested at a more advanced growth stage as petite greens. The variation of NO_3_^-^ concentration observed in the present study may be attributed to the different NO_3_^-^ accumulation capability of the genotypes examined. All of the microgreens examined were grown in a soilless system within the same environment, using a uniform and inert growing medium, and supplied with the same nutrient solution with a constant level of N and other macroelements. The relatively high NO_3_^-^ levels observed suggest that the amount of N provided through the nutrient solution exceeded the N requirement and could be reduced for most of the microgreen’s species examined through tailor made nutrient solution recipes. Despite emerging evidence of the potential beneficial effects of vegetable NO_3_^-^ on human health (Hord et al., 2009; Jonvik et al., 2016), NO_3_^-^ are considered anti-nutrients and the commercialization of several vegetables which are considered a primary source of NO_3_^-^ in the human diet are subject to regulations and restrictions that define maximum limits for NO_3_^-^ content (Di Gioia et al., 2013). Microgreens examined in the present study contained NO_3_^-^ levels that fall within the limits set by the European Commission (EC Reg. No. 1258/2011). Given that the daily consumption of fresh microgreens rarely exceeds 100 g, the contribution of microgreens to the NO_3_^-^ dietary intake of an adult with a regular diet may be relevant, but even with a frequent consumption of microgreens it is unlikely to exceed the reference dose of 1.6 mg of NO_3_-N kg^-1^ bw day^-1^ (equivalent to 7.0 mg NO_3_^-^ kg^-1^ body weight per day) set by the USA Environmental Protection Agency (USA-EPA). Instead, for microgreen species characterized by very high NO_3_^-^ concentration it could be possible to exceed the recommended acceptable daily intake of 3.7 mg NO_3_^-^ kg^-1^ body weight per day set by the Joint Expert Committee of the Food and Agriculture (JECFA) of the United Nations/World Health Organization (WHO) (Mensinga et al., 2003; EFSA, 2008).

**Table 3:** Macroelement and sodium concentration of seventeen microgreen species.^1^

Genotype	N	P	K	Ca	Mg	S	Na
	mg 100 g^-1^ FW						
‘Garnet red’ amaranth	361.02 ± 7.8 bc	62.79 ± 2.6 ab	496.01 ± 11.6 a	141.81 ± 7.4 a	65.16 ± 2.8 a	37.59 ± 1.4 e	6.63 ± 0.33 def
Scallion	421.26 ± 4.8 a	49.58 ± 1.6 cde	317.13 ± 15.1 b	143.73 ± 4.7 a	38.30 ± 1.1 b	68.32 ± 2.4 bcd	3.56 ± 0.07 fgh
‘Black oil’ sunflower	286.07 ± 7.4 ef	66.05 ± 2.1 a	101.31 ± 2.7 f	41.76 ± 1.1 g	39.01 ± 1.3 b	28.30 ± 1.2 efg	3.79 ± 0.58 fgh
Borage	212.30 ± 4.6 h	56.26 ± 0.8 abc	187.50 ± 17.1 def	42.68 ± 1.7 g	18.51 ± 0.3 ef	14.99 ± 0.7 h	9.67 ± 0.75 abcd
Arugula	288.63 ± 4.7 ef	40.93 ± 2.7 ef	316.47 ± 50.0 b	75.27 ± 5.8 bcdef	16.47 ± 1.0 f	64.48 ± 3.0 cd	5.38 ± 0.92 efg
Broccoli	267.64 ± 21.6 efg	37.55 ± 6.5 fg	132.62 ± 28.3 ef	63.20 ± 9.7 defg	19.15 ± 3.2 ef	69.17 ± 11.7 bcd	8.39 ± 1.85 bcde
Red cabbage	312.40 ± 4.1 de	48.23 ± 0.9 cde	279.19 ± 5.2 bc	142.66 ± 11.2 a	30.26 ± 2.1 cd	81.79 ± 6.6 a	12.64 ± 1.40 a
Cressida Cress	278.56 ± 6.3 efg	50.72 ± 0.4 cde	223.56 ± 30.4 cd	50.47 ± 5.3 fg	21.64 ± 0.3 ef	60.23 ± 0.9 d	8.30 ± 0.98 bcde
‘Red Russian’ kale	259.27 ± 9.5 fgh	55.37 ± 2.3 bc	123.49 ± 23.8 f	69.54 ± 3.5 cdefg	25.00 ± 0.6 de	76.97 ± 2.7 ab	8.07 ± 0.60 cde
Mizuna ‘America’	340.05 ± 2.3 cd	48.25 ± 1.1 cde	341.34 ± 16.3 b	101.64 ± 4.6 b	23.96 ± 1.2 de	60.92 ± 0.6 d	12.72 ± 2.51 a
‘Garnet giant’ mustard	291.92 ± 7.7 ef	44.09 ± 4.5 def	214.47 ± 34.3 cde	81.20 ± 10.1 bcde	25.43 ± 2.6 de	58.14 ± 4.1 d	9.19 ± 0.12 bcd
‘Red arrow’ radish	275.98 ± 7.0 efg	54.25 ± 0.7 bcd	104.45 ± 7.1 f	43.95 ± 2.9 g	24.61 ± 0.6 de	75.40 ± 2.5 abc	10.22 ± 0.82 abc
‘Bull’s blood’ beet	276.93 ± 3.3 efg	40.91 ± 1.3 ef	501.47 ± 34.5 a	56.20 ± 4.1 efg	35.60 ± 1.2 bc	20.55 ± 0.7 fgh	11.52 ± 0.39 ab
‘Dark opal’ basil	230.57 ± 7.5 gh	28.35 ± 2.0 g	296.41 ± 20.5 bc	92.27 ± 8.9 bcd	18.77 ± 1.6 ef	18.46 ± 0.5 gh	2.62 ± 0.38 gh
‘Genovese’ basil	282.94 ± 58.7 ef	36.10 ± 9.1 fg	347.79 ± 75.3 b	141.67 ± 32.0 a	29.75 ± 5.7 cd	24.32 ± 2.8 fgh	3.83 ± 0.49 fgh
Britton Shiso	379.43 ± 1.3 abc	56.01 ± 1.9 abc	339.75 ± 12.5 b	96.25 ± 3.9 bc	29.72 ± 1.5 cd	21.96 ± 1.1 fgh	0.69 ± 0.01 h
Lemon Balm	393.19 ± 2.9 ab	57.73 ± 0.9 abc	542.69 ± 2.5 a	89.91 ± 1.3 bcd	36.68 ± 1.1 b	32.42 ± 1.8 ef	3.52 ± 0.54 fgh
*P-value*	*0.0001*	*0.0001*	*0.0001*	*0.0001*	*0.0001*	*0.0001*	*0.0001*

^1^ Data reported are means ± standard error of three replications. Means followed by different letters within each column are significantly different (P < 0.05) by Duncan’s multiple range test. Microgreens were grown under controlled environmental conditions in a polycarbonate greenhouse located in Fort Pierce, FL, USA. Each species was grown in separate growing channels using BioStrate-Felt growing mats and were fertigated with a standard nutrient solution containing only macronutrients.

Among all macroelements, K concentration showed the largest variation ranging from a minimum of 101.3 mg 100 g^-1^ FW in sunflower to a maximum of 542.7 mg 100 g^-1^ FW in lemon balm (**Table 3**). Considering a recommended daily intake of 2,700-4,700 mg of K (World Health Organization, 2003; Stallings et al., 2019), microgreens examined in this study could contribute from a minimum of 2.2% up to 20% of the referenced dietary intake for an adult. Among the microgreens examined at least 8 species were characterized by a K concentration above 300 mg 100 g^-1^ FW and could be considered a good source of K.

Calcium was the third most abundant macro-element and ranged between a maximum of 143.73 mg 100 g^-1^ FW in scallion and a minimum of 41.76 mg 100 g^-1^ FW in sunflower. Considering an estimated daily average requirement of 800 mg of Ca, microgreens examined in this study could contribute from a minimum of 5.2% up to 18% of the reference dietary intake for an adult. Scallion, red cabbage, amaranth, and Genovese basil microgreens could be considered a good source of Ca as a daily portion of 100 g of fresh greens would provide more than 15% of the reference dietary intake for an adult.

Phosphorous concentration was on average 49.01 mg 100 g^-1^ FW and ranged between 28.35 and 66.05 mg 100 g^-1^ FW in basil (green and red) and sunflower microgreens respectively. Considering a recommended daily intake of 700 mg of P, none of the microgreen’s species examined could be considered a good source of P. Following P, Mg content ranged between 16.47 mg 100 g^-1^ FW in arugula and 65.16 mg 100 g^-1^ FW in ‘Garnet red’ amaranth and was on average 29.30 mg 100 g^-1^ FW across all the species examined. Considering an estimated daily average requirement of 350 mg of Mg, only amaranth could provide over 15% of the recommended daily requirement and could be considered the best source of Mg among all the microgreen species considered in the study. Examining the variation of S content, *Brassicaceae* microgreens along with scallion had higher S content (ranging on average between 58.13 and 81.79 mg 100 g^-1^ FW) compared to all the other species studied (ranging on average between 14.99 and 37.59 mg 100 g^-1^ FW). Such results are consistent with those of previous studies and with the fact that species belonging to the *Allium* genus and the *Brassicaceae* family are rich sources of organosulfur compounds and tend to accumulate relatively high levels of S (Petropoulos et al., 2017; Di Gioia et al., 2018; Di Gioia et al., 2019b; Petropoulos et al., 2020; Di Gioia and Petropoulos, 2021).

Finally, as a meso-element Na content was on average 7.10 mg 100 g^-1^ FW and ranged between 0.69 and 12.71 mg 100 g^-1^ FW in Britton Shiso and Mizuna ‘America’, respectively. Sodium is considered an antinutrient and the results of this study suggest that overall microgreens have a relatively low concentration of Na and could be particularly suitable for consumers that must adhere to low Na diets. Nevertheless, it is important to consider that deionized water was used to prepare the nutrient solution in this study, and the content of Na probably could be higher if higher levels of Na were present in the nutrient solution or the growing media (Di Gioia et al., 2017a; Di Gioia et al., 2018).

### 3.3 Variation of micromineral concentration

Among microminerals, the most abundant was Fe followed by Zn, Mn, B, and Cu (**Table 4**). Iron content was on average 0.37 mg 100 g^-1^ FW and ranged between a minimum of 0.25 mg 100 g^-1^ FW in ‘Dark Opal’ basil microgreens and a maximum of 0.47 mg 100 g^-1^ FW in scallion microgreens, suggesting a discrete variation of Fe content across the genotypes tested. These values could be considered relatively low compared to the Fe content reported by Xiao et al. (2016); Di Gioia et al. (2019a), and Kyriacou et al. (2021a) for *Brassicaceae* microgreens. However, in the present study, microgreens were grown on BioStrate growing mats without supplying any micronutrient fertilizer, whereas in the studies of Xiao et al. (2016) and Kyriacou et al. (2021a), microgreens were grown on peat-based media and were fertilized, while in the study of Di Gioia et al. (2019a) microgreens were grown on BioStrate growing mats and also received additional fertilization. In addition to genotype, growing media, fertilization, and other environmental factors can significantly influence the mineral profile of microgreens (Kopsell et al., 2014; Di Gioia et al., 2017a; Weber, 2017). For species other than *Brassicaceae* microgreens there is limited information on the content of Fe and other micronutrients. However, for sunflower microgreens, Poudel et al. (2023a) observed values of Fe in shoots obtained from untreated seeds that were similar to those observed in the present study. Bulgari et al. (2017) observed Fe levels much higher than in the present study for basil and Swiss chard, and Corrado et al. (2021) observed Fe levels over three times lower than in the present study for borage microgreens on a DW basis. Overall, considering an RDA (Recommended Dietary Allowance) for Fe of 8–18 mg per day for adults older than 18 years old and 27 mg per day for pregnant women, relying only on the Fe available through seeds, without fertilizer supplementation, the contribution of microgreens to reaching the Fe RDA would be relatively low across all the species tested (NRC, 1989).

**Table 4:** Microelement concentration of seventeen microgreen species.^1^

Genotype	Fe	Zn	B	Mn	Cu
	mg 100 g^-1^ FW				
‘Garnet red’ amaranth	0.432 ± 0.03 abc	0.297 ± 0.02 efg	0.137 ± 0.01 cdef	0.109 ± 0.002 ef	0.070 ± 0.003 ef
Scallion	0.473 ± 0.05 a	0.484 ± 0.04 b	0.371 ± 0.02 a	0.333 ± 0.025 a	0.104 ± 0.005 c
‘Black oil’ sunflower	0.446 ± 0.07 ab	0.749 ± 0.03 a	0.161 ± 0.01 bc	0.157 ± 0.005 cd	0.239 ± 0.009 a
Borage	0.411 ± 0.004 abcd	0.397 ± 0.01 cd	0.078 ± 0.01 h	0.095 ± 0.004 f	0.093 ± 0.001 d
Arugula	0.303 ± 0.01 de	0.294 ± 0.01 efgh	0.102 ± 0.01 efgh	0.062 ± 0.001 g	0.035 ± 0.003 l
Broccoli	0.336 ± 0.03 bcde	0.340 ± 0.02 ef	0.129 ± 0.01 cdefg	0.109 ± 0.01 ef	0.037 ± 0.001 il
Red cabbage	0.321 ± 0.02 cde	0.247 ± 0.004 ghi	0.113 ± 0.01 defgh	0.119 ± 0.005 ef	0.035 ± 0.003 l
Cressida Cress	0.318 ± 0.04 cde	0.404 ± 0.01 c	0.141 ± 0.01 cde	0.155 ± 0.008 cd	0.049 ± 0.001 gh
‘Red Russian’ kale	0.414 ± 0.03 abcd	0.308 ± 0.01 ef	0.102 ± 0.01 efgh	0.110 ± 0.01 ef	0.034 ± 0.002 l
Mizuna ‘America’	0.363 ± 0.01 abcde	0.315 ± 0.01 ef	0.102 ± 0.01 efgh	0.114 ± 0.005 ef	0.053 ± 0.003 gh
‘Garnet giant’ mustard	0.343 ± 0.05 bcde	0.213 ± 0.02 i	0.116 ± 0.01 defgh	0.105 ± 0.006 f	0.035 ± 0.004 l
‘Red arrow’ radish	0.346 ± 0.02 bcde	0.346 ± 0.01 de	0.089 ± 0.02 gh	0.120 ± 0.005 ef	0.052 ± 0.004 gh
‘Bull’s blood’ beet	0.398 ± 0.04 abcd	0.239 ± 0.01 ghi	0.101 ± 0.01 efgh	0.245 ± 0.002 b	0.060 ± 0.003 fg
‘Dark opal’ basil	0.254 ± 0.02 e	0.282 ± 0.01 fgh	0.120 ± 0.01 defg	0.053 ± 0.002 g	0.047 ± 0.002 hi
‘Genovese’ basil	0.333 ± 0.06 bcde	0.236 ± 0.02 hi	0.146 ± 0.01 cd	0.065 ± 0.007 g	0.065 ± 0.007 f
Britton shiso	0.452 ± 0.01 ab	0.320 ± 0.004 ef	0.098 ± 0.003 fgh	0.165 ± 0.005 c	0.119 ± 0.003 b
Lemon balm	0.311 ± 0.01 cde	0.246 ± 0.01 ghi	0.186 ± 0.02 b	0.136 ± 0.006 de	0.078 ± 0.002 e
*P-value*	*0.004*	*0.0001*	*0.0001*	*0.0001*	*0.0001*

^1^ Data reported are means ± standard error of three replications. Means followed by different letters within each column are significantly different (P < 0.05) by Duncan’s multiple range test. Microgreens were grown under controlled environmental conditions in a polycarbonate greenhouse located in Fort Pierce, FL, USA. Each species was grown in separate growing channels using BioStrate-Felt growing mats and were fertigated with a standard nutrient solution containing only macronutrients.

Zinc content was on average 0.34 mg 100 g^-1^ FW and ranged between a minimum of 0.21 mg 100 g^-1^ FW in ‘Garnet Giant’ mustard and a maximum of 0.75 mg 100 g^-1^ FW in sunflower microgreens, which indicates a large variation in Zn content across the genotypes tested. For *Brassicaceae* microgreens, the range of Zn concentrations was consistent with that observed in previous studies (Xiao et al., 2016; Di Gioia et al., 2019a; Kyriacou et al., 2021a). The relatively high content of Zn observed in sunflower microgreens was consistent with the values observed by Poudel et al. (2023a) in sunflower shoots derived by untreated seeds. In the case of borage, the Zn concentration observed in the present study was over four times higher than the concentration observed by Corrado et al. (2021) for the same species on a DW basis. Taking into account a Zn RDA of 2 and 5 mg for infants and pre-school children and up to 8–11 mg per day for adult females and males, respectively, all microgreens tested could be considered a good source of Zn for children, but inadequate for adults (NRC, 1989). In fact, except for sunflower, most of the microgreen species tested could provide only a small fraction of the Zn RDA for adults without supplementary Zn fertilization, although biofortification is a possible route to increase its Zn content (Poudel et al., 2023a).

Given that large portions of the global population suffer from the deficiency of both Fe and Zn, implementing agronomic biofortification strategies to increase the content of both essential micronutrients may substantially increase the content of Fe and Zn in microgreens (Di Gioia et al., 2019a; Poudel et al., 2023a) and the results of the present study allow for the identification of those species that tend to accumulate more Fe and Zn.

Examining the content of Mn and Cu, their content was consistent with the range of concentrations observed by Xiao et al. (2016) and Di Gioia et al. (2019a) in *Brassicaceae* species, while Kyriacou et al. (2021a) reported much higher Mn content for four *Brassica rapa* L. microgreens. For sunflower, Poudel et al. (2023b) reported values of Mn and Cu slightly lower than those observed in the present study on a DW basis. On the other hand, Corrado et al. (2021) reported higher levels of Mn and much lower levels of Cu in borage microgreens compared to the levels observed in this study. When considering the RDA of Mn (1.8–2.3 mg per day for adult females and males, respectively) and Cu (1 mg per day for adult women and men), among the species examined in the present study, only scallion and sunflower could be considered a good source of Mn and Cu, respectively. Therefore, for Mn and Cu supplementing microminerals through the application of nutrient solutions during their relatively short growth cycle may enhance their mineral profile and increase their contribution to achieving the RDA of different microminerals.

Boron content was on average 0.34 mg 100 g^-1^ FW and ranged between a minimum of 0.08 mg 100 g^-1^ FW in borage and a maximum of 0.18 mg 100 g^-1^ FW in lemon balm microgreens, except for scallion microgreens that had a much higher B content (0.37 mg 100 g^-1^ FW) compared to all the other species. Very limited information is available in the literature on the variation of B content in microgreens, but in the case of sunflower, similar levels were observed by Poudel et al. (2023b) in sunflower shoots derived from untreated seeds. Conversely, D’Imperio et al. (2021) observed B levels nearly three times higher in mizuna microgreens grown in peat and fertilized with half-strength Hoagland nutrient solution. Boron deficiency is not prevalent in humans and an intake of 1-3 mg/day for an adult is considered a sufficient daily intake range; therefore, scallion microgreens could be considered a good source of B.

### 3.4 Principal component analysis

The PCA performed on the normalized data revealed four principal components (PCs) with eigenvalues >1 that explained approximately 81% of the total variance in the data set.

**Figure 4:**
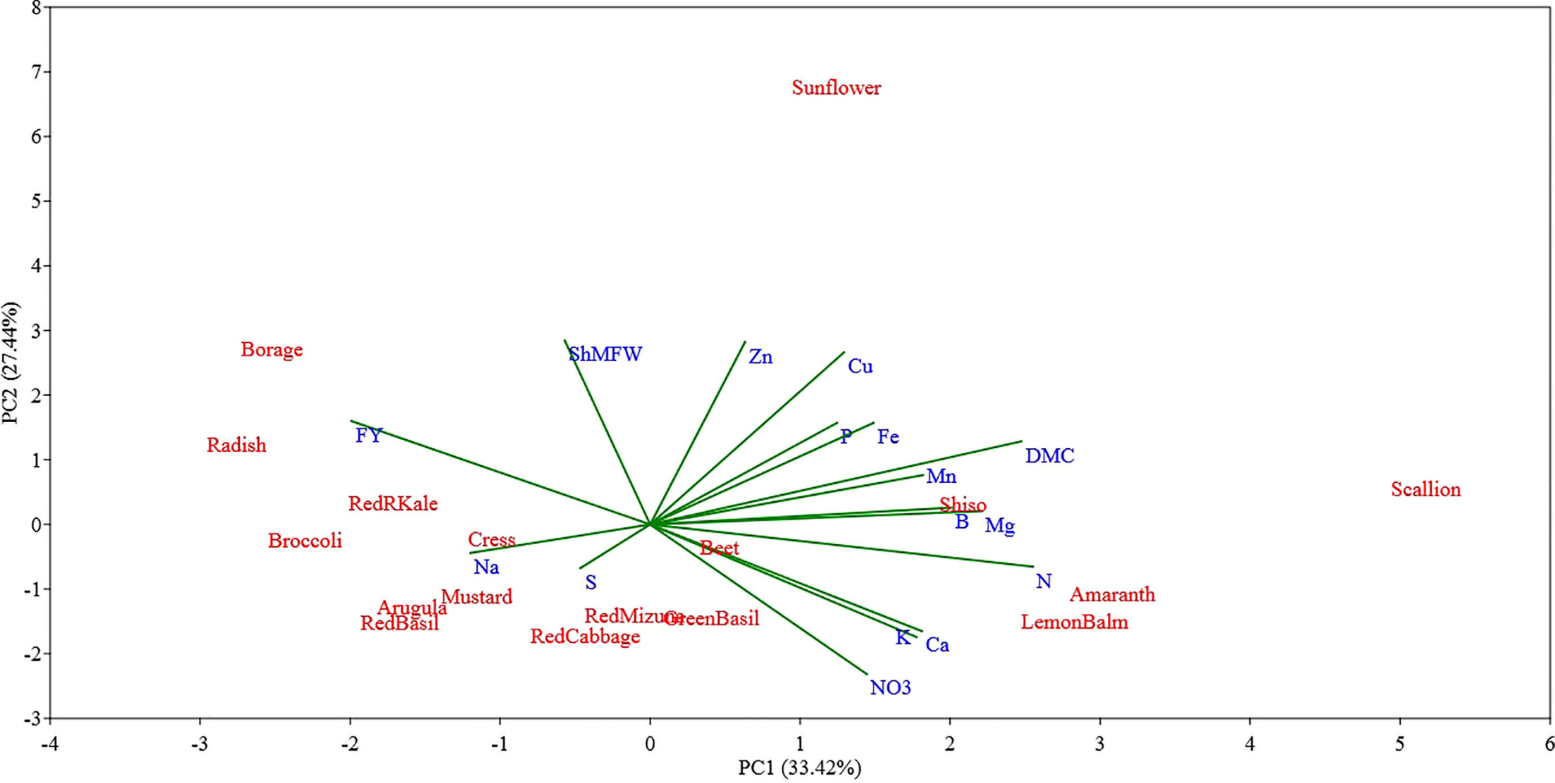
Principal component (PC) analysis biplot (PC1 versus PC2) showing the spatial distribution of the mineral profile and yield component of seventeen selected microgreens belonging to seven botanical families and grown in a soilless system under controlled environment. Parameters considered include fresh yield (FY), shoot mean fresh weight (ShMFW), dry matter content (DMC) and the concentration of minerals: total N, P, K, Ca, Mg, S, Na, Fe, Zn, Mn, Cu, B, and nitrate (NO_3_^-^).

The PC1-PC2 bidimensional graph presented in **Figure 4** show a clear distinction between the different microgreen species examined for their biometric parameters and mineral profile. The first two principal components (PCs) accounted for 60.86% of the total variance, attributing 33.42% to PC1 and 27.44% to PC2, respectively. Most of the variables examined were positively correlated with PC1, and only a few parameters such as fresh yield, shoot mean fresh weight, S and Na were negatively correlated with PC1. The variables with the highest positive correlation coefficient were N (0.88) and dry matter content (0.85), followed by Mg (0.76), B (0.69), Mn (0.63), Ca (0.62), and K (0.61) content. The PC1 was negatively correlated with microgreen fresh yield (-0.68), Na content (-0.41), shoot mean fresh weight (-0.20), and S (-0.16) content. The PC1 allowed separation of the seventeen microgreen species based on their yield potential which was apparently negatively correlated to microgreen N content and dry matter content.

The PC2 was positively correlated with shoot mean fresh weight (0.89), Zn (0.88) and Cu (0.83) content, and fresh yield (0.50), and it was negatively correlated with the content of NO_3_^-^ (-0.72), K (-0.54), and Ca (-0.51). The PC2 allowed separation of the species examined based on their single shoot mean fresh weight and their Zn, Cu, and NO_3_^-^ content.

**Figure 5:**
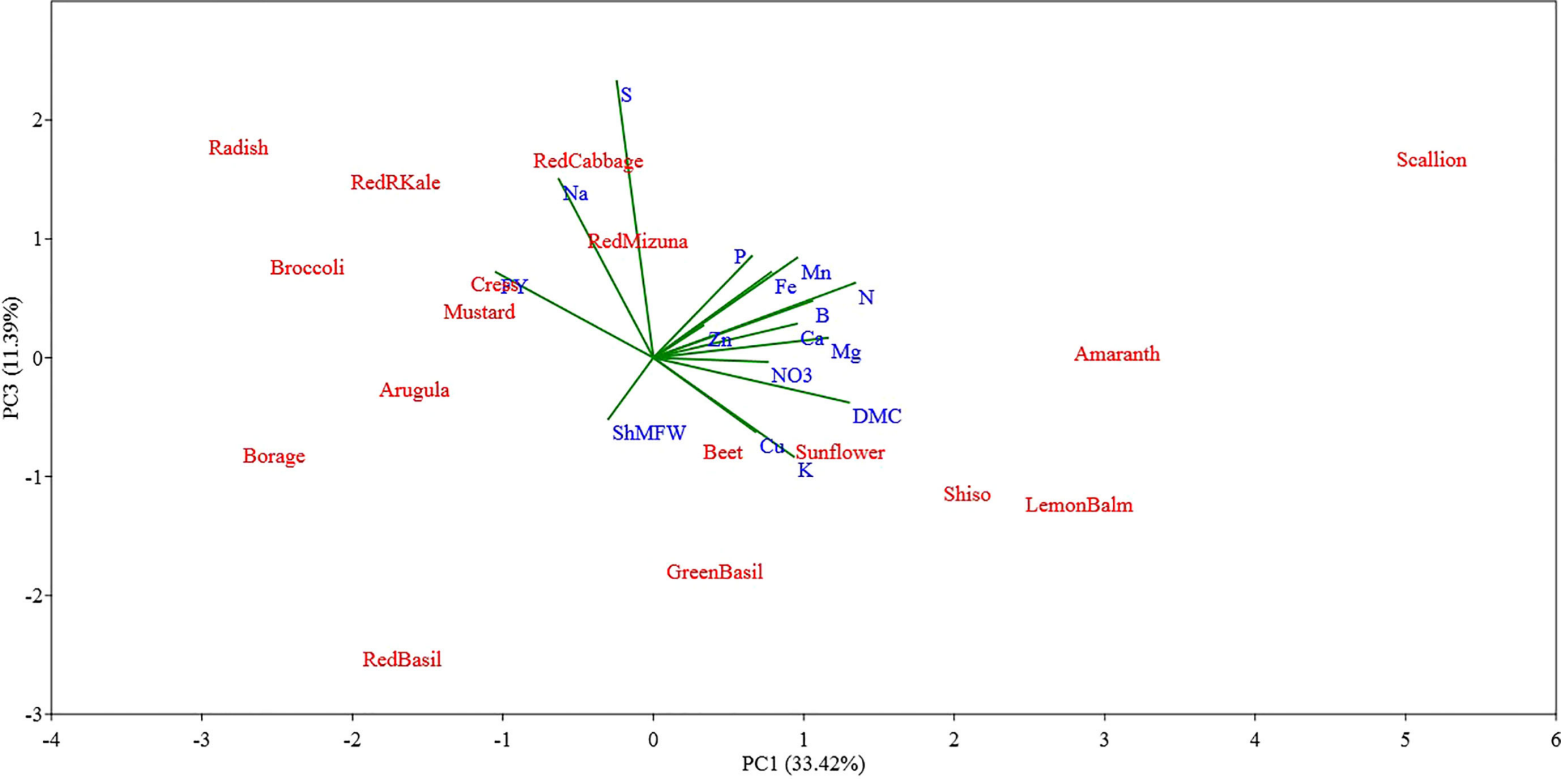
Principal component (PC) analysis biplot (PC1 versus PC3) showing the spatial distribution of the mineral profile and yield component of seventeen selected microgreens belonging to seven botanical families and grown in a soilless system under controlled environment. Parameters considered include fresh yield (FY), shoot mean fresh weight (ShMFW), dry matter content (DMC) and the concentration of minerals: total N, P, K, Ca, Mg, S, Na, Fe, Zn, Mn, Cu, B, and nitrate (NO_3_^-^).

**Figure 6:**
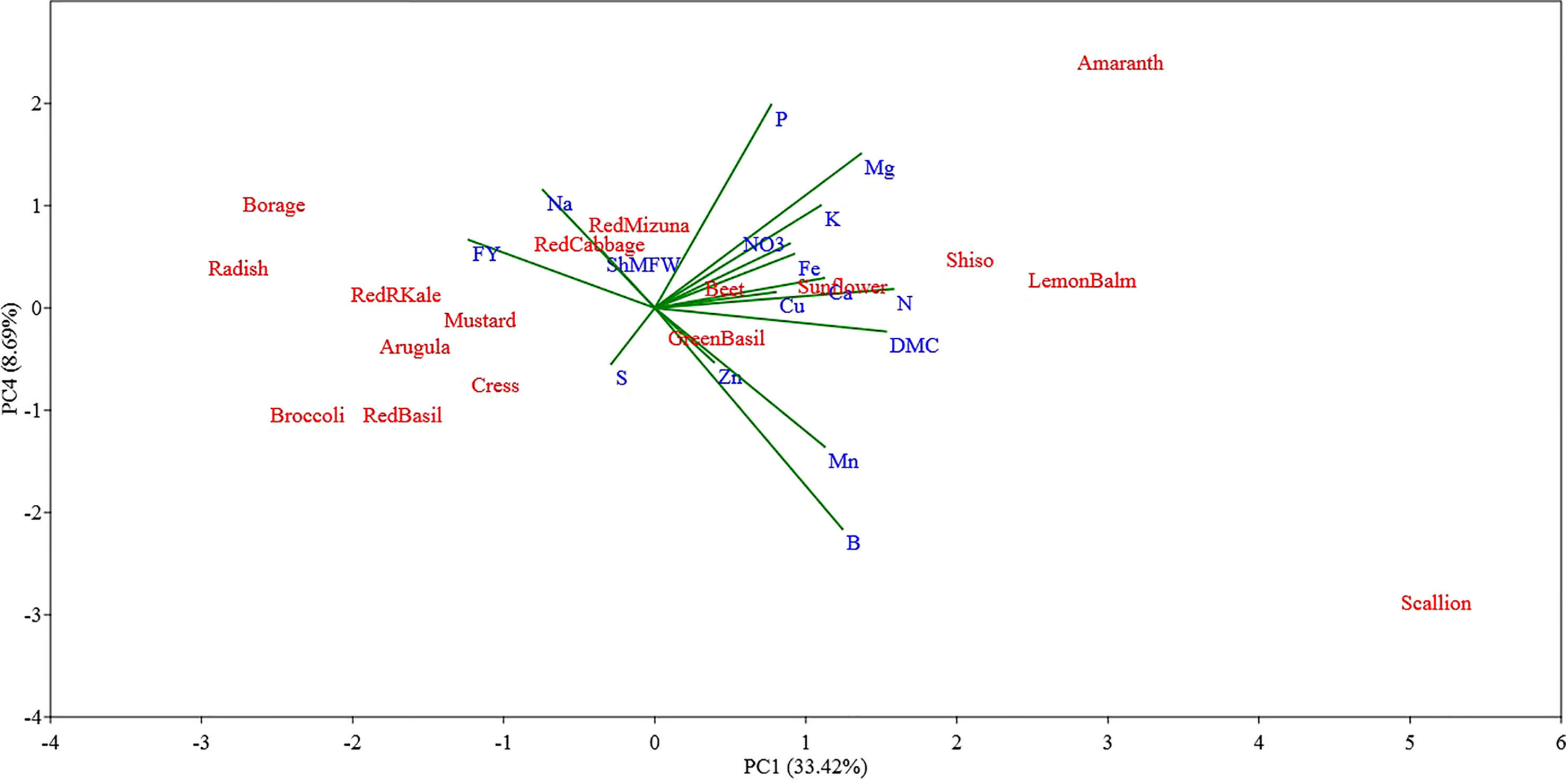
Principal component (PC) analysis biplot (PC1 versus PC4) showing the spatial distribution of the mineral profile and yield component of seventeen selected microgreens belonging to seven botanical families and grown in a soilless system under controlled environment. Parameters considered include fresh yield (FY), shoot mean fresh weight (ShMFW), dry matter content (DMC) and the concentration of minerals: total N, P, K, Ca, Mg, S, Na, Fe, Zn, Mn, Cu, B, and nitrate (NO_3_^-^).

The PC3 and PC4 explained 11.39% and 8.69% of the total variance in the data set, respectively (**Figures 5**, **6**). The PC3 was positively correlated with S (0.66) and Na (0.43); while PC4 was positively correlated with P (0.48) and Mg (0.36) and was negatively correlated with B (-0.52).

Examining the first two PCs, the PCA analysis revealed clear distinctions between scallion and sunflower from all other species, as well as between themselves (**Figure 4**). Scallion was strongly associated with DMC, Mg, N, and Mn, and located at the first quadrant with a high PC1 value and low PC2 value. On the other hand, sunflower was strongly associated with Zn, Cu, P, and Fe, and located at the first quadrant with a low PC1 value and high PC2 value. Most *Brassicaceae* species were grouped together with two basils (*Lamiaceae*) in or near the third quadrant, and associated with Na, S, and FY. Borage, separated from other species, was located at the second quadrant with a moderate absolute PC1 value and low PC2 value, and was strongly associated with FY. Radish, kale, and broccoli were relatively close to borage, and they were also strongly associated with FY. Shiso and Lemon balm, along with amaranth, were clustered together in the borderline area between the first and fourth quadrants with moderate PC1 values and low PC2 values, and were associated with DMC, Mn, B, Mg, N, Ca, K, and NO_3_. Beet, located near the center of the PCA plot, indicating a neutral mineral balance in the plant (**Figure 4**). The PC3 being correlated with S and Na separated all of the tested *Brassicaceae* species and scallion, characterized by relatively high S and Na content, also separated from all other species, particularly from red and green basil which were characterized by low S and Na content (**Figure 5**). The PC4 separated species based on their content of B, P, and Mg primarily, and scallion, which was characterized as having high B content, was located in the fourth quadrant, and amaranth, that was associated with relatively high content of P and Mg, was located in the first quadrant.

## 4 Conclusions

Based on the present study, it is apparent that the yield potential and mineral profile of microgreens are largely determined by the selected genotype. Fresh yield was on average 1,175.7 g m^-2^ and ranged from 409.3 g m^-2^ for lemon balm up to 2,258.8 g m^-2^ in the case of radish. A positive correlation was observed between single shoot mean fresh weight and the average seed weight, suggesting that the size of shoots is determined at least in part by the average seed weight. Examining the mineral profiles of the seventeen species selected, there was significant genetic variation observed for all of the minerals analyzed. Nitrogen and K were the principal macronutrients accounting on average for 38.4% and 33.8% of the total macroelement concentration, respectively, followed in order by Ca, P, S, and Mg. Taking into consideration the recommended dietary allowance (RDA) of different minerals, several microgreen species provided more than 15% of the RDA and could be considered as a good source of one or more essential minerals. Among microminerals, the most abundant was Fe followed by Zn, Mn, B, and Cu. Considering that no microminerals were provided through the nutrient solution, the content of micromineral was relatively low for all the species tested and could be associated with the initial seed reserves. Nevertheless, sunflower, scallion, and shiso could be considered as good sources of Cu and sunflower was a good source of Zn. Overall, microgreens can be considered a good source of minerals, and using selections belonging to different botanical families, it is possible to obtain edible products that are richer or less rich in specific minerals, while the wide variety of mineral profiles could be used to address different consumer dietary needs and diversify dietary sources on a daily basis.

## Data availability statement

The original contributions presented in the study are included in the article/supplementary material. Further inquiries can be directed to the corresponding author.

## Author contributions

FD and ER developed the idea, designed, and coordinated the study. FD, JH, CP, ER conducted the experiment. ER provided the resources to conduct the experiment. FD managed and analyzed the data, interpreted the results, and wrote the first draft of the manuscript. ER, SAP, JH, CP, and JB, contributed to edit the manuscript. All authors edited the manuscript and approved the submitted version.
